# Relationship Between Flow and Absolute Humidity During Active Humidification for High-Flow Therapy: A Bench Study

**DOI:** 10.3390/medsci14050588

**Published:** 2026-09-19

**Authors:** Riccardo Re, Sergio Lassola, Eleonora Balzani, Marco Parisi, Michele Zeni, Giacomo Bellani

**Affiliations:** 1 Department of Anesthesia and Intensive Care 1, Santa Chiara Hospital, ASUIT, Largo Medaglie d’Oro 9, 38122 Trento, Italy; sergio.lassola@asuit.tn.it (S.L.); eleonara.balzani@asuit.tn.it (E.B.); marco.parisi1@asuit.tn.it (M.P.); michele.zeni@asuit.tn.it (M.Z.); giacomo.bellani@asuit.tn.it (G.B.); 2 Centre for Medical Sciences (CISMed), University of Trento, Via S. Maria Maddalena 1, 38122 Trento, Italy

**Keywords:** humidification, High Flow Nasal Cannula, High Flow Oxygen Therapy, Continuous Positive Airway Pressure, absolute humidity, temperature, intensive care units

## Abstract

Active humidification of medical gases is a fundamental component of high-flow oxygen therapy (HFOT). Given the high gas flow rates employed, it remains unclear whether the theoretically expected relationship between flow rate, temperature, and absolute humidity is maintained under clinical conditions. This bench-top study aimed to characterize the relationship between absolute humidity (AH) and gas flow rate and to identify optimal AH targets across different HFOT settings. This relationship was assessed by measuring water consumption by the heated humidifier at different temperatures and across a range of high-flow settings. Statistical analysis showed that AH increased by approximately 3.7 mg/L for each 1 °C increase in temperature and decreased by approximately 0.4 mg/L for each additional 1 L/min of delivered flow. Utilizing the alveolar absolute humidity value of 44 mg/L at 37 °C as the reference, the derived slope was used to estimate the corresponding AH targets for each high-flow therapy setting: 10.4 mg/L for Continuous Positive Airway Pressure via Helmet (H-CPAP), 25.3 mg/L for High Flow Nasal Cannula (HFNC), and 36.5 mg/L for HFOT-tracheostomy. Given the decrease in absolute humidity with increasing gas flow rate, the study also proposes a temperature-compensation model that may be used to achieve target absolute humidity values across different high-flow therapy settings.

## 1. Introduction

Active humidification of medical gases is a fundamental component of the management and optimization of both invasive and non-invasive ventilation [1,2]. Adequate humidification prevents a range of complications that may compromise patient safety and the efficacy of ventilatory support [1,3]. Inadequate humidification and heating of medical gases can lead to bronchial mucosal dryness, resulting in epithelial injury, impaired mucociliary clearance, altered mucus rheology, and increased susceptibility to respiratory infections. During invasive ventilation, insufficient humidification may also increase the risk of endotracheal tube obstruction and hypothermia [4]. Conversely, excessive humidification or overheating of inspired gases may cause mucosal burns, secretion accumulation, and impaired mucociliary function [4,5,6]. Both inadequate and excessive humidification may also affect surfactant production, promoting micro-atelectasis and consequently reducing functional residual capacity and pulmonary compliance [7].

**Figure 1:**
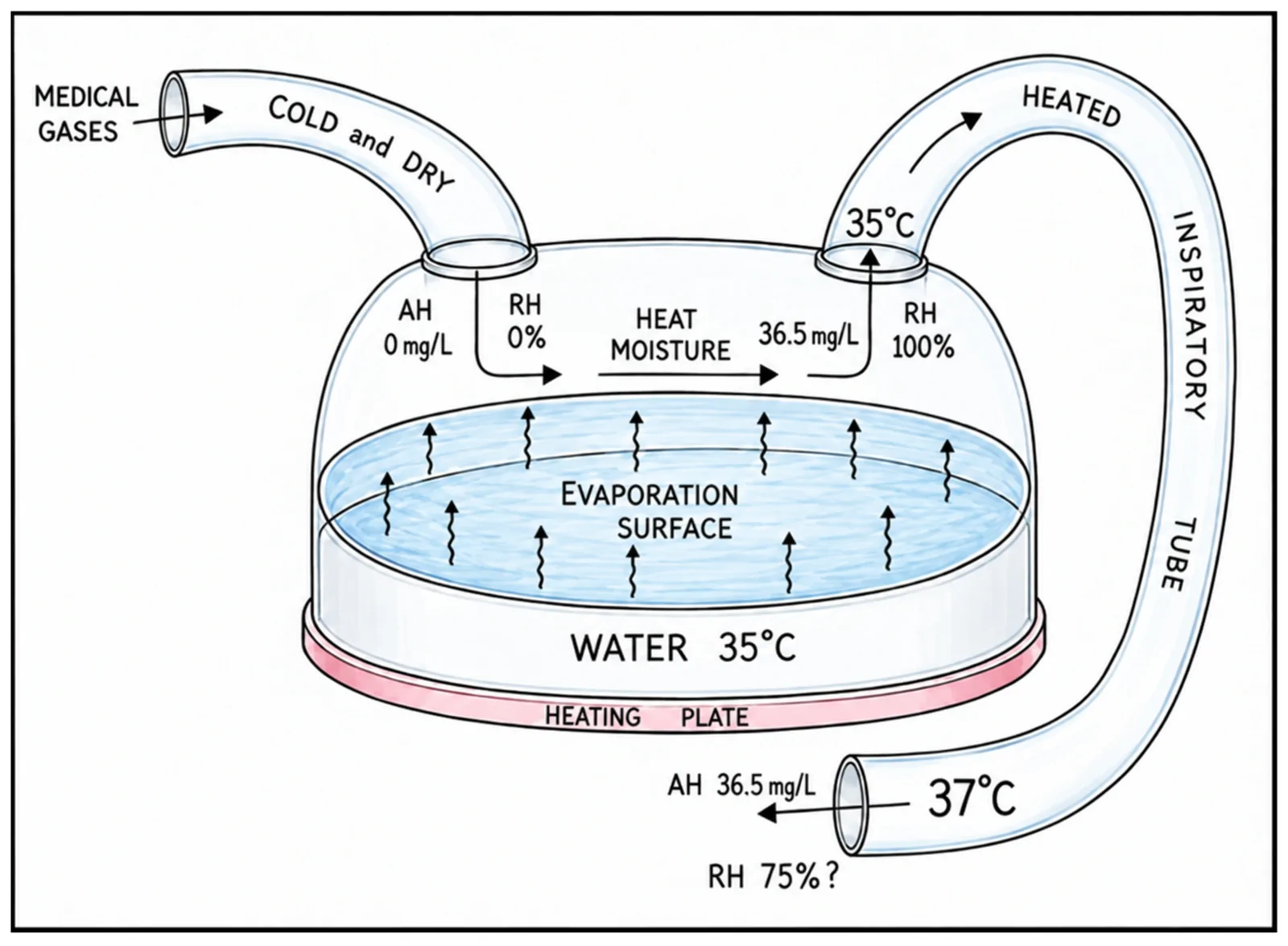
Graphical representation of the humidification process within a passover heat humidifier (p-HH). Medical gas enters the humidifier cold and dry. Heating of the water promotes evaporation, thereby increasing the gas temperature and delivering water vapor until the gas reaches the desired temperature and 100% relative humidity (RH), with a corresponding absolute humidity (AH). To prevent condensation within the inspiratory circuit, the tubing must be heated, which increases gas temperature, reduces RH, and increases the capacity of the gas to carry water vapor. This process allows the intended AH to be maintained and delivered to the patient.

Heating and humidification of inspired gases are commonly achieved using active humidifiers. Among these, passover heat humidifiers (p-HHs) are widely used in intensive and sub-intensive care settings. These systems incorporate a heated water chamber, a heated inspiratory circuit, and temperature sensors, allowing precise control of gas temperature and humidity. Their ability to provide fine adjustments while closely mimicking the physiological process of airway humidification has contributed to their widespread use [8]. The absolute humidity delivered by these devices results from the evaporation of water within the humidification chamber, generating water vapor in the gaseous phase without entrained liquid droplets [9]. The principles governing the humidification of medical gases by p-HHs are based on the close interrelationship between temperature (T), absolute humidity (AH), and relative humidity (RH). Heating the water within the humidifier chamber induces evaporation, thereby determining the amount of water vapor introduced into the gas stream. The amount of water present in the gaseous phase is defined as AH and is expressed in mg/L. To ensure adequate humidification of medical gases and the effective delivery of water vapor to the patient, while preventing condensation within the inspiratory circuit, relative humidity must be carefully considered. Relative humidity is defined as the ratio, expressed as a percentage, between the actual amount of water vapor present in a gas and the maximum amount of water vapor that the gas can hold at a given temperature and pressure. At the outlet of the humidifier chamber, the gas is saturated and close to the dew point, corresponding to an RH of 100%. To prevent condensation on the cooler surfaces of the breathing circuit and ensure delivery of the intended AH to the patient, the inspiratory tubing must be heated along its length. The resulting increase in gas temperature lowers the RH and increases the capacity of the gas to hold water vapor, thereby preventing condensation [8,9] (Figure 1).

Reference temperature targets for different ventilation modes are well established in the literature. The additional humidity supplied by the HH is intended to condition the inspired gas to achieve the physiological alveolar gas humidity of 44 mg/L of AH at 100% RH [1,2,8,9]. Recommended chamber temperatures are approximately 28 °C for non-invasive ventilation delivered via a helmet or mask, 32 °C for High Flow Nasal Cannula (HFNC) therapy, and 35 °C for invasive ventilation and High Flow Oxygen Therapy-tracheostomy (HFOT-t) [8,10,11,12,13]. With respect to the temperature targets for high-flow therapies, flow rates typically range from 30 to 60 L/min for HFNC [14,15,16,17] and from 60 to 100 L/min for Continuous Positive Airway Pressure delivered via a helmet (H-CPAP) [3,12,18,19,20,21]. Similarly, invasive HFOT-t generally employs flow rates within the same range as those used for HFNC [22,23]. In this process, temperature acts as the primary controllable variable, whereas gas flow represents an independent parameter. From a physical standpoint, under constant temperature and contact surface area, the rate of water evaporation is directly proportional to the gas flow rate through the humidifier chamber. Thus, higher gas flow rates should theoretically enhance evaporation and maintain a stable level of AH generated by the p-HH [8,9]. Despite these well-established physical principles, the clinical use of such high flow rates raises questions as to whether the theoretical relationship between gas flow, temperature, and absolute humidity is effectively maintained under real-world conditions.

The primary aim of this study is to characterize the relationship between AH and gas flow, with the main hypothesis that an increase in gas flow does not result in a reduction in the AH of the delivered medical gases. A secondary aim is to identify the potential optimal AH targets across different HFOT settings. To address this secondary endpoint, the data analysis will allow determination of the T/AH relationship and the corresponding slope, thereby identifying target humidity values for each of the ventilation modalities investigated. These targets will be derived using the physiological alveolar AH of 44 mg/L at 37 °C as the reference value previously reported in the literature [1,2,6,7,8,9,10].

## 2. Materials and Methods

Gas flow was generated using a turbine-driven ventilator (Hamilton C6, Hamilton Medical, Bonaduz, Switzerland) operating in High Flow mode, with a flow sensitivity of 1 L/min and an initial automatic flow calibration. Flow rates were set at 30, 40, 50, 60, 80, and 100 L/min according to the experimental phase. The fraction of inspired oxygen (FiO_2_) was maintained at 100% to ensure a constant initial humidity of the source gas, as pressurized oxygen from the centralized supply is completely free of water vapor. This condition prevented variability that could otherwise arise from the entrainment of ambient air by the turbine, as ambient air contains variable levels of water vapor depending on environmental humidity. Active humidification was provided using a passover humidifier (Dräger Aquapor H300, Drägerwerk AG, Lübeck, Germany), with a temperature setting sensitivity of 0.5 °C. No specific calibration of the device was performed before testing, as the equipment undergoes regular maintenance and calibration by the manufacturer.

During the humidification of medical gases using a p-HH, AH represents the most clinically relevant measure of gas humidity. Therefore, in the present study, AH was determined indirectly from the amount of water consumed by the humidifier rather than measured using conventional capacitive hygrometers, which typically measure RH. Although AH can be measured directly using a chilled-mirror hygrometer, this type of equipment was not used in the present study because of its high cost and limited availability. Accordingly, AH was calculated as the ratio between the volume of water consumed by the humidifier and the volume of gas delivered by the system, expressed in mg/L.

### 2.1. Study Protocol

**Figure 2:**
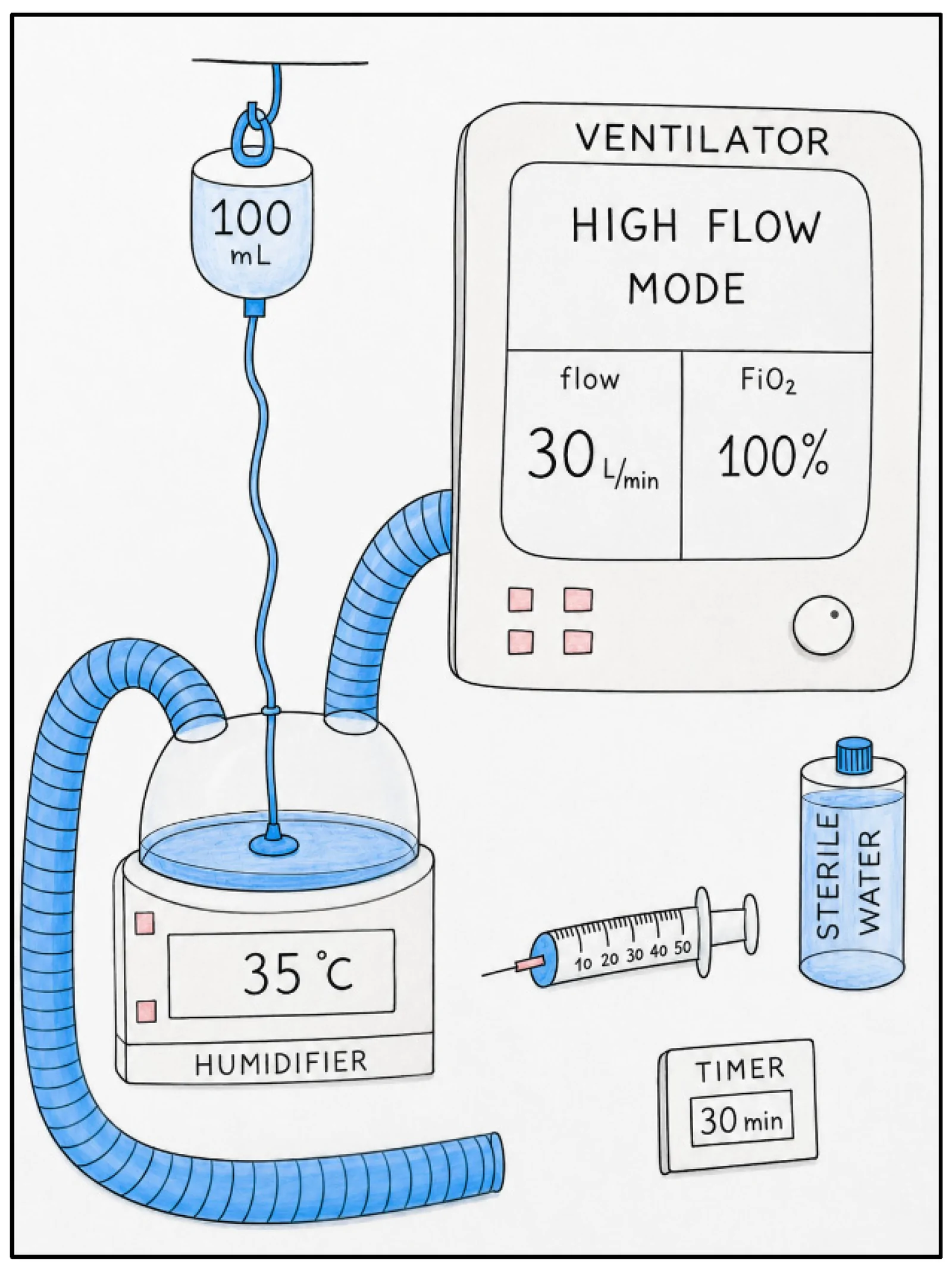
Illustration of the bench study.

Prior to each individual test, the humidifier chamber was filled with sterile distilled water using a self-regulating drip set. Once the preset temperature for the specific experimental condition had been reached, as verified directly using the dedicated temperature-check function on the humidifier, the standard water supply bag was replaced with an intravenous bag containing 100 mL of distilled water, and a 30 min timer was started. At the end of the 30 min period, the volume of water remaining in the bag was measured using a 50 mL syringe with 1 mL graduation (BD Plastipak™, Becton Dickinson S.A., San Agustin del Guadalix, Madrid, Spain). Water consumption was calculated as the difference between the initial volume (100 mL) and the remaining volume, corresponding to the volume of water evaporated during the 30 min test period. The total volume of gas delivered during each test was calculated from the preset flow rate and the test duration. Assuming a density of distilled water of 997 kg/m^3^, the volume of evaporated water was converted to mass and used to calculate the AH for each temperature–flow combination. AH was calculated as the ratio of the mass of evaporated water to the total volume of gas delivered and expressed in mg/L (Figure 2).

To simulate the realistic clinical scenarios, the following 11 combination were tested, Table 1:

Temperature settings were manually adjusted on the humidifier, and the selected chamber temperature was verified using the device’s dedicated temperature-confirmation function.

### 2.2. Statistical Analysis

Continuous variables were expressed as mean ± standard deviation (SD) or median [25, 75 interquartile range (IQR)], as appropriate.

The association between AH and the experimental variables was initially assessed using Pearson’s correlation coefficients, with corresponding *p*-values. As a sensitivity analysis, Spearman’s rank correlation coefficients were also calculated; these results are not reported unless they differed materially from those obtained using Pearson’s correlation.

To quantify the effects of gas flow and chamber temperature on AH, simple linear regression models were performed. These models provided unadjusted regression coefficients (β) with corresponding 95% confidence intervals (CIs).

A multivariable linear regression model including both gas flow and chamber temperature as independent variables was then fitted to estimate their adjusted associations with AH. An interaction term (gas flow × chamber temperature) was added to assess whether the association between gas flow and AH varied across temperature levels. Model fit was assessed using the coefficient of determination (R^2^), Akaike information criterion (AIC), and residual analysis.

All statistical analyses were performed using R version 4.3.1 (R Foundation for Statistical Computing, Vienna, Austria). A two-sided *p*-value < 0.05 was considered statistically significant.

### 2.3. Tests of Normality

Normality was assessed based on model residuals using the Shapiro–Wilk test, together with visual inspection of Q–Q plots. Homoscedasticity was assessed using the Breusch–Pagan test. Residuals from the univariable models were consistent with normality (*p* = 0.81 and *p* = 0.06), whereas residuals from the multivariable model showed evidence of departure from normality (*p* = 0.007), with no evidence of heteroscedasticity (*p* = 0.80). Accordingly, the multivariable model estimates were further evaluated using heteroscedasticity-consistent (HC3) standard errors and a 5000-replicate bootstrap procedure. Both approaches yielded estimates consistent with those obtained using ordinary least squares, supporting the robustness of the regression results. The detailed results are reported in Supplementary Table S1.

## 3. Results

Across all tests performed, the measured temperature remained close to the preset values of 28, 32, and 35 °C, with a maximum deviation of ±0.2 °C.

A total of 33 flow–temperature combinations were tested, and the mean AH was calculated for each combination (Table 2).

### 3.1. Impact of Temperature Settings

**Figure 3:**
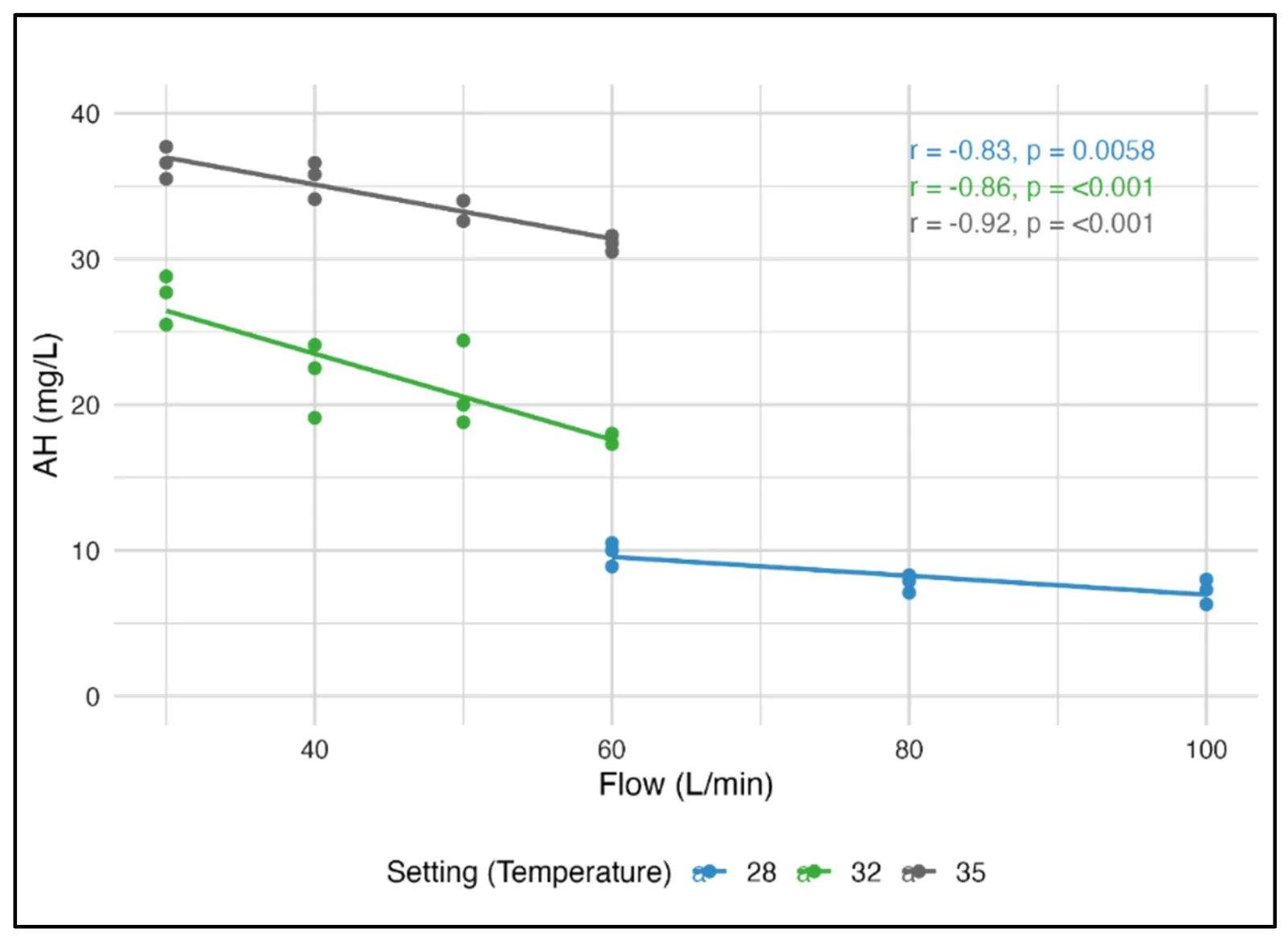
Relationship between absolute humidity (AH, mg/L), gas flow (L/min), and the three humidifiers chamber temperature settings (28, 32, and 35 °C).

At the H-CPAP setting (chamber temperature, 28 °C), a significant negative correlation was observed between AH and gas flow (r ≈ −0.83, *p* = 0.0058). At the HFNC setting (chamber temperature, 32 °C), a strong negative correlation was also observed between AH and gas flow (r ≈ −0.86, *p* < 0.001). Finally, at the HFOT-t setting (chamber temperature, 35 °C), a very strong negative correlation was observed between AH and gas flow (r ≈ −0.92, *p* < 0.001) (Figure 3).

### 3.2. Linear Regression Univariate

The univariable linear regression model assessing the association between AH and chamber temperature was expressed as follows: AH (mg/L) = −95.80 + 3.73 × Temperature (°C), with an intercept of −95.80 (95% CI, −107.80 to −83.81) and a slope of 3.73 (95% CI, 3.33 to 4.08). The model showed a strong positive correlation (r = 0.96), explained 93% of the variability in AH (R^2^ = 0.93), and was statistically significant (*p* < 0.001).

**Figure 4:**
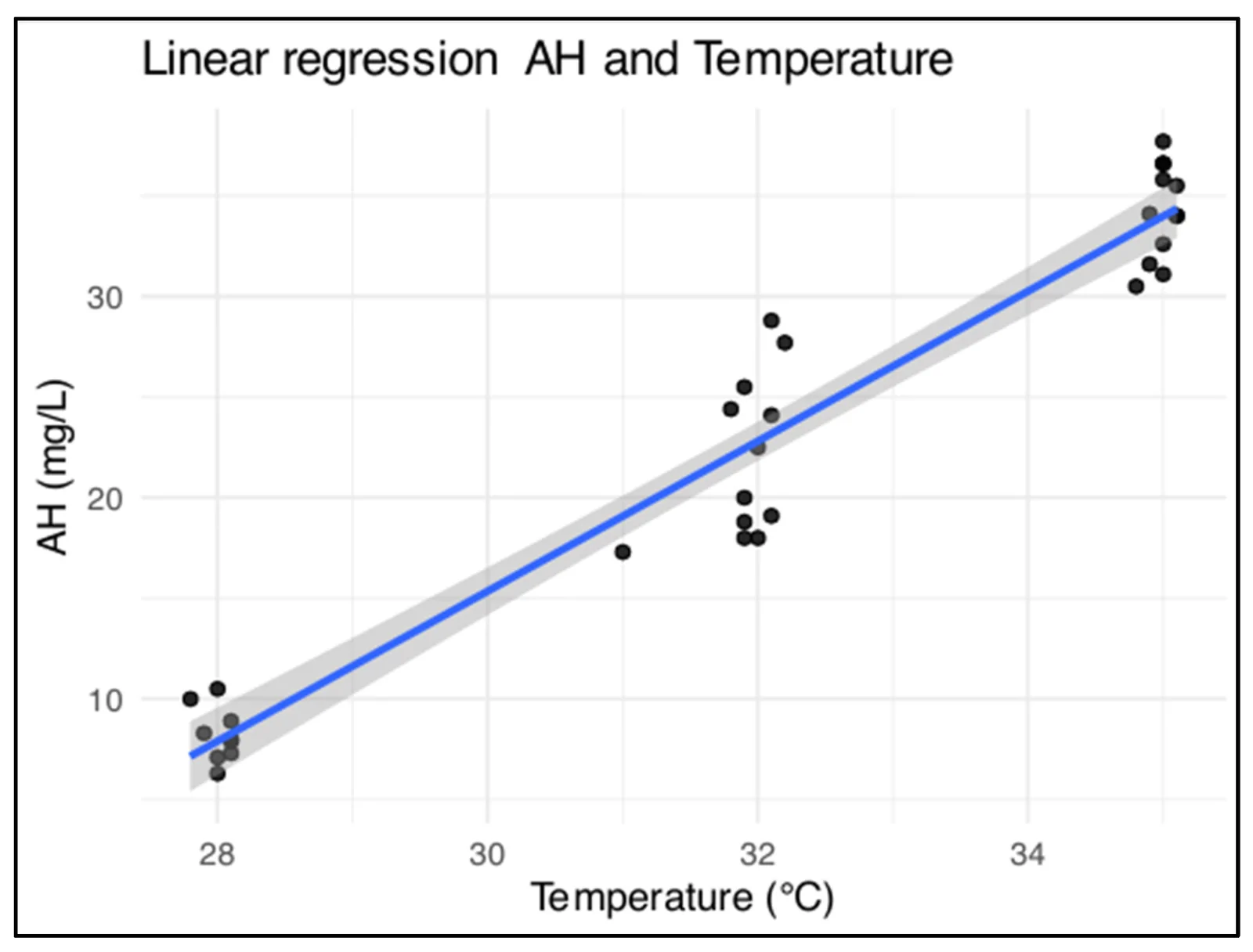
Absolute Humidity (AH) as a function of humidifier chamber temperature across the tested flow–temperature combinations. The blue line represents the fitted linear regression slope.

The univariable linear regression model assessing the association between AH and gas flow was expressed as follows: AH (mg/L) = 44.73 − 0.40 × Flow (L/min), with an intercept of 44.73 (95% CI, 37.55 to 51.91) and a slope of −0.40 (95% CI, −0.53 to −0.28). The model showed a strong negative correlation (r = −0.77), explained 59% of the variability in AH (R^2^ = 0.59), and was statistically significant (*p* < 0.001). Both models were fitted exclusively over the tested ranges (28–35 °C for chamber temperature and 30–100 L/min for gas flow); therefore, the intercepts represent mathematical extrapolations beyond the experimental range and have no direct physical interpretation. Overall, the analysis showed a positive association between AH and chamber temperature, with a slope of 3.73 mg/L per °C increase in temperature (Figure 4). Conversely, AH decreased with increasing gas flow, with a slope of −0.40 mg/L per L/min.

In practical terms, AH increased by approximately 3.7 mg/L for each 1 °C increase in chamber temperature and decreased by approximately 0.4 mg/L for each 1 L/min increase in gas flow.

### 3.3. Multivariable Model Between AH/Flow/Temp

In the multivariable analysis examining the relationship between AH, temperature, and gas flow, chamber temperature was the variable most strongly associated with humidification. After adjustment for gas flow, each 1 °C increase in temperature was associated with an increase of 3.22 mg/L in AH (*p* < 0.0001). Conversely, gas flow showed a weaker negative association with AH; for each 1 L/min increase in gas flow, absolute humidity decreased by 0.0997 mg/L (*p* < 0.001). Overall, the model showed excellent fit (R^2^ = 0.96; AIC = 152).

**Figure 5:**
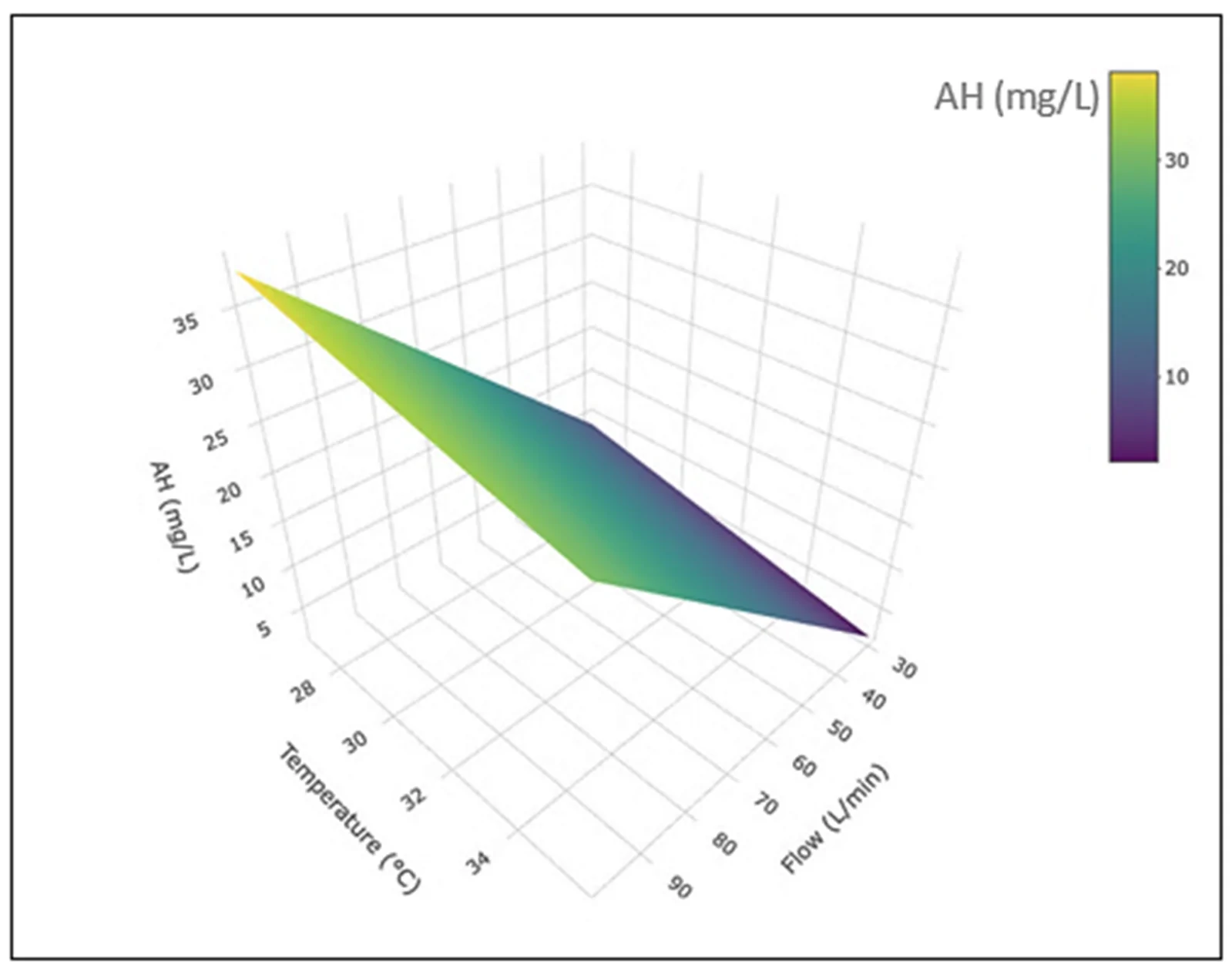
Three-dimensional response surface illustrating the relationship between absolute humidity (AH), chamber temperature, and gas flow. The color gradient from purple to yellow represents increasing AH.

Figure 5 illustrates this relationship through a three-dimensional response surface, where AH is plotted as a function of both temperature and flow. The surface clearly shows that humidification increases steeply with temperature, while rising flow slightly reduces humidity by limiting the time available for gas-water interaction.

### 3.4. AH Targets Determination and Temperature Compensation Model

Using the physiological alveolar AH of 44 mg/L at 37 °C as the reference value and the derived slope of 3.73 mg/L per °C, we calculated the target AH for each chamber temperature setting. The resulting target AH values were 10.4 mg/L for H-CPAP, 25.3 mg/L for HFNC, and 36.5 mg/L for HFOT-t.

On the basis of these findings, we developed a theoretical temperature compensation model suggesting that an increase in chamber temperature may compensate for the reduction in AH associated with increasing gas flow (Table 3). The target AH values derived from this model may differ slightly from those reported in Figure 4, as the latter were based on measurements obtained under the flow conditions applied during the experiments.

## 4. Discussion

The main finding of this bench-top study is that the air temperature within the humidifier chamber is an important factor determining the rate of water evaporation and, consequently, the absolute humidity delivered to the system. The chamber temperature remained within ±0.2 °C of the setpoint throughout the experiments, indicating that the humidifier can adjust its heating resistance in response to variations in flow, while consistently maintaining the target temperature. This mean that temperature, and consequently the rate of water evaporation, is an independent variable. This condition is particularly important, as it indicates that the results obtained in this study are primarily dependent on variations in gas flow.

The results demonstrated a significant inverse relationship between gas flow and delivered AH, with higher flow rates resulting in reduced AH and a diminished ability of the humidifier to maintain optimal humidity levels.

Since the chamber air temperature remained constant across different flow rates, one might expect the humidifier to maintain an adequate rate of water evaporation and, consequently, a constant AH per liter of gas flow. However, this expectation was not supported by the findings of the present study, which, as previously mentioned, demonstrated an inverse relationship between flow rate and AH. This discrepancy may hypothetically be attributed to the onset of a flow-bypass phenomenon, whereby a fraction of the airflow could bypass direct contact with the evaporative surface while being indirectly heated through radiative heat transfer from the humidifier chamber. Under such conditions, the bypassed air would undergo heating without contributing to the humidification process. Nevertheless, this interpretation remains speculative, as the present study did not provide sufficient experimental evidence to confirm the occurrence of a flow-bypass phenomenon within the humidifier chamber. In the absence of direct measurements of airflow distribution, this proposed mechanism should therefore be considered a potential explanation for the observed findings rather than an established physical phenomenon.

Overall, the reduction in AH was modest at low-to-moderate flow rates (30–50 L/min), with a decrease of less than 15%, but became more pronounced at higher flow rates (60–100 L/min), particularly during H-CPAP, where AH decreased by up to 30%. Notably, the inverse relationship between flow rate and AH appeared to be less pronounced at higher temperatures, suggesting a potential hypothetic compensatory interaction between thermal and flow dynamics during the humidification process.

The study also confirmed a linear increase in AH with temperature and a decrease with increasing flow rate, as shown by the univariable model (Figure 4). In the multivariable AH–flow–temperature model, these associations were slightly attenuated but remained statistically significant, demonstrating a temperature-dependent increase in AH and a less pronounced reduction in AH at higher flow rates (Figure 5).

The proposed temperature compensation model (Table 3) integrates previously published data with the novel findings of the present study, thereby providing a more clinically relevant perspective on the bench-based investigation. Although the model is supported by a substantial body of experimental evidence, further validation is warranted to assess its robustness and generalizability across different clinical conditions and settings. The current level of evidence should also be interpreted in light of several methodological limitations of the present study. These include the use of a single type of humidifier, the absence of an independent external dual-control system for measuring humidity, temperature, and flow, and the relatively small sample size. Despite these limitations, the proposed model may represent a valuable starting point for optimizing the humidification of medical gases during high-flow therapies. In particular, the development of humidification systems with adjustable operating parameters could enable dynamic adaptation to the clinically required variations in flow associated with different HFOT modalities. Furthermore, the hypothesized flow-bypass phenomenon warrants further investigation. A more detailed characterization of gas flow patterns within the humidifier chamber could contribute to optimizing gas distribution and improving contact with the evaporative surface. Such design modifications may increase the efficiency of p-HHs while ensuring consistent and adequate humidification of medical gases.

## 5. Conclusions

Despite the limitations inherent to a bench-top study, the present research demonstrated that active humidification during high-flow therapy is strongly influenced by flow rate, with AH decreasing by 0.4 mg/L for each additional L/min of delivered flow. Furthermore, the study identified a linear relationship between chamber temperature and AH, with AH increasing by 3.73 mg/L for each 1 °C increase in temperature. The AH–temperature slope allows the definition of target AH values for different high-flow therapies and provides the basis for a compensation model to adjust humidifier temperature in response to increasing flow rates. However, the proposed temperature compensation model requires further investigation and should be validated in future clinical studies. Nevertheless, it may represent a valuable starting point for further research aimed at optimizing humidification across different HFOT modalities.

## Figures and Tables

**Table 1:** Simulation of realistic clinical scenarios. Continuous Positive Airway Pressure via Helmet (H-CPAP), High Flow Nasal Cannula (HFNC), High Flow Oxygen Therapy-tracheostomy (HFOT-t).

Temperature	Flows	Simulated Clinical Scenarios
28 °C	60, 80, and 100 L/min	H-CPAP
32 °C	30, 40, 50, and 60 L/min	HFNC
35 °C	30, 40, 50, and 60 L/min	HFOT-t

**Table 2:** Flow–temperature combinations and corresponding mean and standard deviation of AH. Continuous Positive Airway Pressure via Helmet (H-CPAP); High Flow Nasal Cannula (HFNC); High Flow Oxygen Therapy-tracheostomy (HFOT-t).

Setting	30 L/min	40 L/min	50 L/min	60 L/min	80 L/min	100 L/min
H-CPAP, 28 °C	-	-	-	9.8 ± 0.8	7.8 ± 0.6	7.2 ± 0.9
HFNC, 32 °C	27.3 ± 1.7	21.9 ± 2.6	21.1 ± 3.0	17.8 ± 0.4	-	-
HFOT-t, 35 °C	36.6 ± 1.1	35.5 ± 1.3	33.5 ± 0.8	31.1 ± 0.6	-	-

**Table 3:** Temperature Compensation Model. Absolute humidity (AH, mg/L), gas flow (L/min), and temperature (Temp, °C). Continuous Positive Airway Pressure via Helmet (H-CPAP); High Flow Nasal Cannula (HFNC); High Flow Oxygen Therapy-tracheostomy (HFOT-t).

Therapy	Temp. Setting	Target AH	Flow	Temp. Compensation
			60 L/min	28 °C
H-CPAP	28 °C	10.4 mg/L	80 L/min	29 °C
			100 L/min	30 °C
			30 L/min	31 °C
HFNC	32 °C	25.3 mg/L	40 L/min	33 °C
			50 L/min	33 °C
			60 L/min	35 °C
			30 L/min	35 °C
HFOT-t	35 °C	36.5 mg/L	40 L/min	35.5 °C
			50 L/min	36 °C
			60 L/min	37.5 °C

## Data Availability

The original contributions presented in this study are included in the article/Supplementary Material. Further inquiries can be directed to the corresponding author.

## Supplementary Materials

The following supporting information can be downloaded at: https://www.mdpi.com/article/10.3390/medsci14050588/s1, Table S1: Test of normality.
