# Supplementary material for: Relationship Between Flow and Absolute Humidity During Active Humidification for High-Flow Therapy: A Bench Study
Source: Med Sci (Basel). 2026 Sep 19;14(5):588. doi: 10.3390/medsci14050588 (PMC13609765; doi:10.3390/medsci14050588)
Supplement: Supplementary file 1 [file medsci-14-00588-s001.zip › medsci-4513658-supplementary.pdf]

**Model**	**Shapiro-Wilk**	**Breusch-Pagan**
AH ~ Temperature	p = 0.805	p = 0.426
AH ~ Flow	p = 0.055	p = 0.626
AH ~ Temperature + Flow	p = 0.007	p = 0.803

Table S1

Supplementary Table S1. Results of the tests of normality performed on the residuals of the univariable and multivariable regression models.
